# Light absorption enhancement in thin film GaAs solar cells using dielectric nanoparticles

**DOI:** 10.1038/s41598-022-13418-4

**Published:** 2022-06-02

**Authors:** Fateh A. Chaudhry, Lorena Escandell, Eduardo López-Fraguas, Ricardo Vergaz, José Manuel Sánchez-Pena, Braulio García-Cámara

**Affiliations:** GDAF-UC3M, Dep. Tecnología Electrónica, Universidad Carlos III de Madrid, Avda. Universidad, 30, 28911 Leganés, Madrid Spain

**Keywords:** Solar energy, Nanophotonics and plasmonics, Metamaterials

## Abstract

Cost-effective and lightweight solar cells are currently demanded in strategic fields such as space applications or integrated-wearable devices. A reduction of the active layer thickness, producing thin-film devices, has been a traditional solution to accomplish both requirements. However, this solution also reduces the efficiency of the device. For this reason, alternative strategies are being proposed. In this work, light trapping effects of an array of semiconductor nanoparticles located on the top surface of a thin-film GaAs solar cell are investigated to improve the optical absorption and current density in active layer, under the standard AM-1.5 solar spectrum. The numerical results are compared with other previous proposals such as an aluminum nanoparticle array, as well as conventional solar cells with and without a standard anti-reflective coating (ARC). The inclusion of semiconductor nanoparticles (NPs) shows an improved response of the solar cells at different angles of incidence in comparison to solar cell with an ARC. Furthermore, the efficiency increases a 10% respect to the aluminum nanoparticles (NPs) architecture, and a 21% and a 30% respect to solar cells with and without ARC, respectively.

## Introduction

Solar energy is one of the most relevant and world spread sustainable sources with a mature related technology. Consequently, it should be part of the solution to current energetic and climatic problems. However, solar cell technology should still adequate its characteristics to market requirements. In this way, the present market demands cost-effective and lightweight devices with remarkable power conversion efficiency to provide a massive expansion of their use, as well as its integration in mobile and wearable devices^1^.

Solar devices of the so-called second generation are also known as thin-film devices because of the reduced dimensions of their active layer. Unfortunately, a reduction of the semiconductor material not only involves a decrease of the cost and the weight but also a dramatic decrease of the conversion efficiency. The consequent generation, the third one, came to solve this issue from different approaches^2–5^. Multi-junction devices with several stacked active layers working at different and complementary spectral ranges, or new materials (e.g., perovskites) are some of the successful alternatives^6,7^. Additionally, the control of light arose as an interesting strategy to increase the solar cell efficiency by maximizing the amount of light within the active layer. Following this idea, optical elements such as textured electrodes^8^, integrated Bragg reflectors^9^, diffractive gratings^10,11^, photonic crystals^12^, resonant nanostructures^13,14^ or nanoparticles producing up-conversion effects^15^ have been included. In this framework, resonant nanoparticles can confine light into a sub-wavelength volume and scatter it with a certain directional control. For this reason, their inclusion in solar cells has been being analyzed since several years ago^15,16^. Depending on the targeted dominant effect, these nanoparticles may be placed on the top, inside or at the bottom part of the devices reducing both reflection and parasitic losses, and increasing photon absorption. While several works have been mainly focused on plasmonic nanoparticles^17–19^, their ohmic losses and low thermochemical stability^20^ may reduce the lifetime of the device. In contrast, dielectric resonant nanostructures are recently being considered^21–23^.

Moreover, while silicon has still a dominant position in the photovoltaic industry, other different materials are also in a mature position for the fabrication of solar cells. This is the case of gallium arsenide (GaAs) which presents efficiencies comparable to silicon in single crystalline devices. Additionally, its interesting properties, like its high photoelectric conversion efficiency per mass density, make it suitable for thin-film solar cells^24^. Other relevant properties such as its low temperature coefficient and radiation resistance make it also an excellent material for space and high-altitude platforms^25^.

In this work, we demonstrate the successful operation of a thin-film GaAs solar cell that includes resonant dielectric nanoparticles on the top surface. These nanoparticles are placed with the aim of reducing the surface reflection but also to efficiently scatter light into the device. A joint effect between the Mie resonances of dielectric nanoparticles and the diffractive modes of the arrangement provides the expected management of the incident light. A numerical optimization process has been carried out, involving both the material and the geometrical properties of the nanostructures, as well as carefully analyzing the spectral profiles of the total reflectance, absorption rate and the short-circuit current density. The considered optimal device shows a remarkable enhancement of the solar cell performance, with a relative increase larger than 20% compared with other solutions used in the state of the art, such as an antireflection coatings (ARC)^26,27^ and plasmonic nanoparticles^28–30^. Moreover, the considered geometry has been designed with the intention of ease the fabrication in contrast to other complex shapes like nanopyramids or nanocones.

## Setup and methods

**Figure 1:**
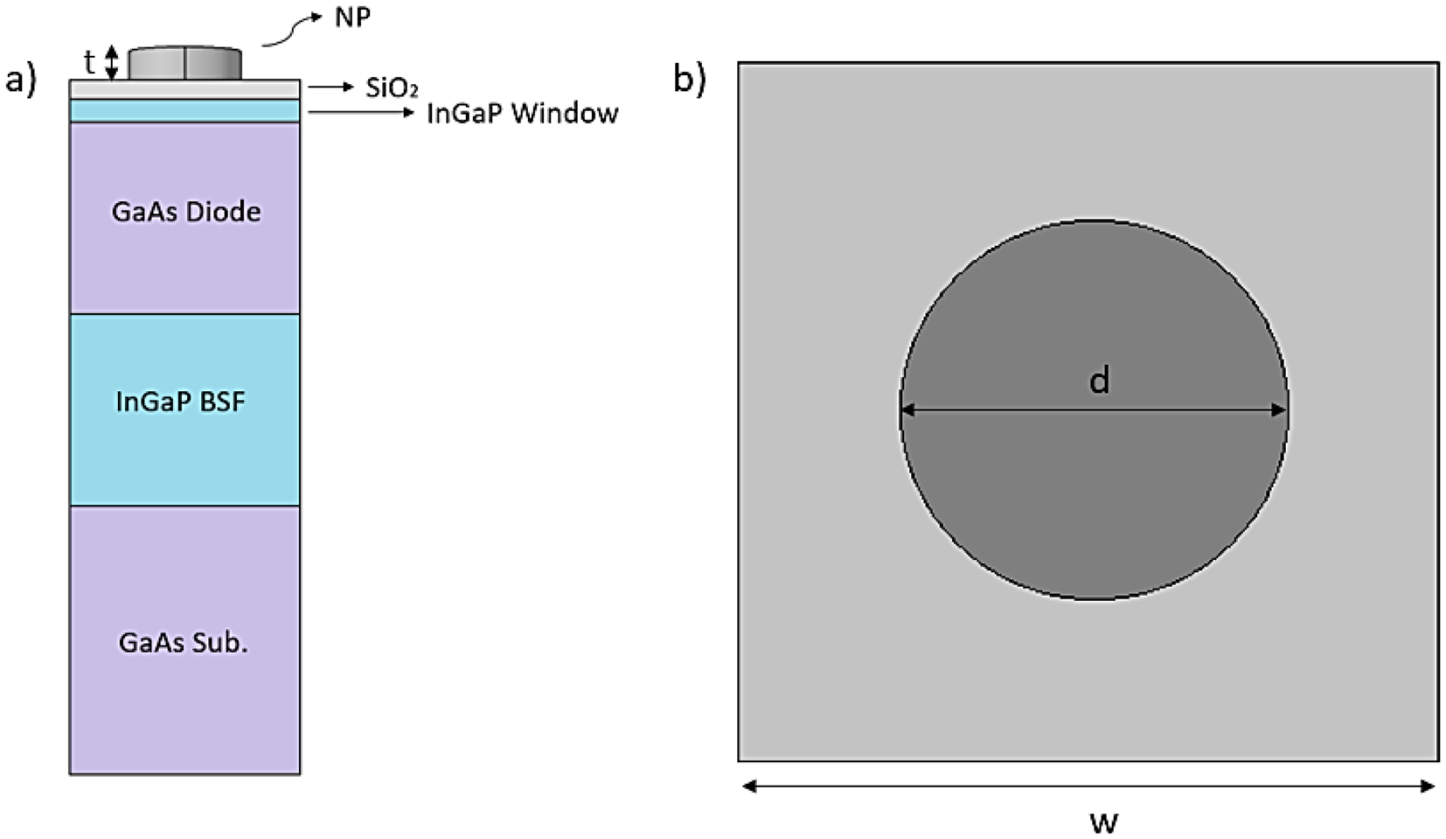
(**a**) Scheme of the proposed GaAs solar cell with dielectric nanoparticles on the front surface. (**b**) Top view of the device, including labels of the main considered geometrical parameters.

A unit cell of the proposed device is shown in Fig. 1. This is a gallium arsenide (GaAs) solar cell, which arrangement, materials, and geometrical parameters are similar to those considered in previous works^31,32^. The detailed description of the different layers can be found in Ref. 32, where experimental samples were characterized. This structure has been considered to provide a convenient reference which efficiency has been also evaluated using plasmonic nanoparticles^31,32^. In our particular case, the top surface includes resonant dielectric nanoparticles to improve the light trapping inside the solar device.

The different component layers are the following ones, from top to bottom: cylindrical dielectric nanoparticles (NP) on the top, which diameter (d), thickness (t) and material will be optimized in this work, a silicon dioxide (SiO_2_) layer of 25 nm acting as a space layer, an indium gallium phosphide (InGaP) window layer of 30 nm, a GaAs active layer of 500 nm, a InGaP back-surface-field (BSF) layer of 500 nm, and a GaAs substrate with a large thickness in comparison to the other layers. In order to find an optimal optical operation, the period (w) of the nanoparticles array will be also varied.

It is important to remark that a realistic solar device requires the insertion of other layers, such as those n- and p-doped layers generating such a suitable electric field as to separate the photogenerated carriers^32^. However, their thicknesses and low refractive index contrasts make them negligible in an optical analysis as the one that we present here. Nevertheless, we previously checked that this was not an issue by using the refractive indexes from reference^33^ and inserting both layers, not having any effect in the results to be presented from here on. For this reason, they are not included in this simplified structure.

We use the finite element method (FEM) implemented by COMSOL Multiphysics © to simulate the optical behavior of the device and the absorption rate of each layer. We set periodic boundary conditions (see unit cell at Fig. 1a) to model a periodic array of the unit cell while perfect matched layers are set at the top and bottom absorbing boundaries. The different media are considered isotropic, with their actual complex refractive indices obtained from SOPRA database^34^.

The internal (IQE) and external (EQE) quantum efficiencies are common parameters to characterize photovoltaic devices. It is also widely accepted in optical analysis of these devices as the one presented here to consider an IQE of 100%. This means that each absorbed photon is supposed to create one electron–hole pair. Consequently, the optical performance of the device through this assumption retrieves a value of the EQE that must be considered as a maximum one.

Another way to characterize the performance of the device is by calculating the short-circuit current density (J_SC_) in the active layers, by using the following integration over the spectral range.

1 $$ J_{SC} = \int {q\frac{\lambda }{hc}WDA\left( \lambda \right) \cdot P_{AM1.5} \left( \lambda \right) \cdot d\lambda } $$

where q is the electron charge, c is the speed of light in vacuum, h is the Planck’s constant, and P_AM1.5_ is the standard solar incident irradiance AM1.5G (1000 W/m^2^). The wavelength-dependent absorbance, WDA(λ) has been calculated through the power loss function. To compute it, the absorbed power density in each layer of the device, and particularly in the active layer, is divided by the input power, and then integrated over the layer volume.

Finally, and for the sake of comparison, we also consider two other systems: a GaAs solar cell with the same geometrical characteristics and materials composition as the previous one but without any light-trapping technique, and a GaAs solar cell as this latter one, including a typical antireflection coating (ARC) made of magnesium fluoride (MgF_2_) and zinc sulfide (ZnS). This allows us to contrast our results with those references, evaluating the achievements of the proposed system in comparison to other well-known configurations^31^.

## Results and discussion

As it was previously stated, the aim of this work is to maximize the optical performance of a conventional GaAs solar cell by taking advantage of the light resonances of dielectric nanoparticles located on top of it. To optimize this arrangement, we previously made a search for the adequate geometrical properties; these are the height (t), diameter (d), and array period (w) of the nanoparticles. Additionally, different dielectric materials have been also considered. After this recursive analysis and comparing its results in terms of absorption and reflection with those from previous works^31^, we observed that the geometrical properties providing the best results within the solar spectrum are a diameter (d) of 250 nm, a height (t) of 50 nm and a period w = 350 nm. Regarding the material of the nanoparticles, the results limit the choice to two different dielectric materials: TiO_2_ and AlAs, due to their valuable results. For this reason, hereinafter only those two dielectric materials are considered. In the last years, there can be found several ways to properly fabricate this kind of ordered arrays in an accurate way, therefore the geometrical dimensions feasibility is in accordance with the state of the art^35–37^.

**Figure 2:**
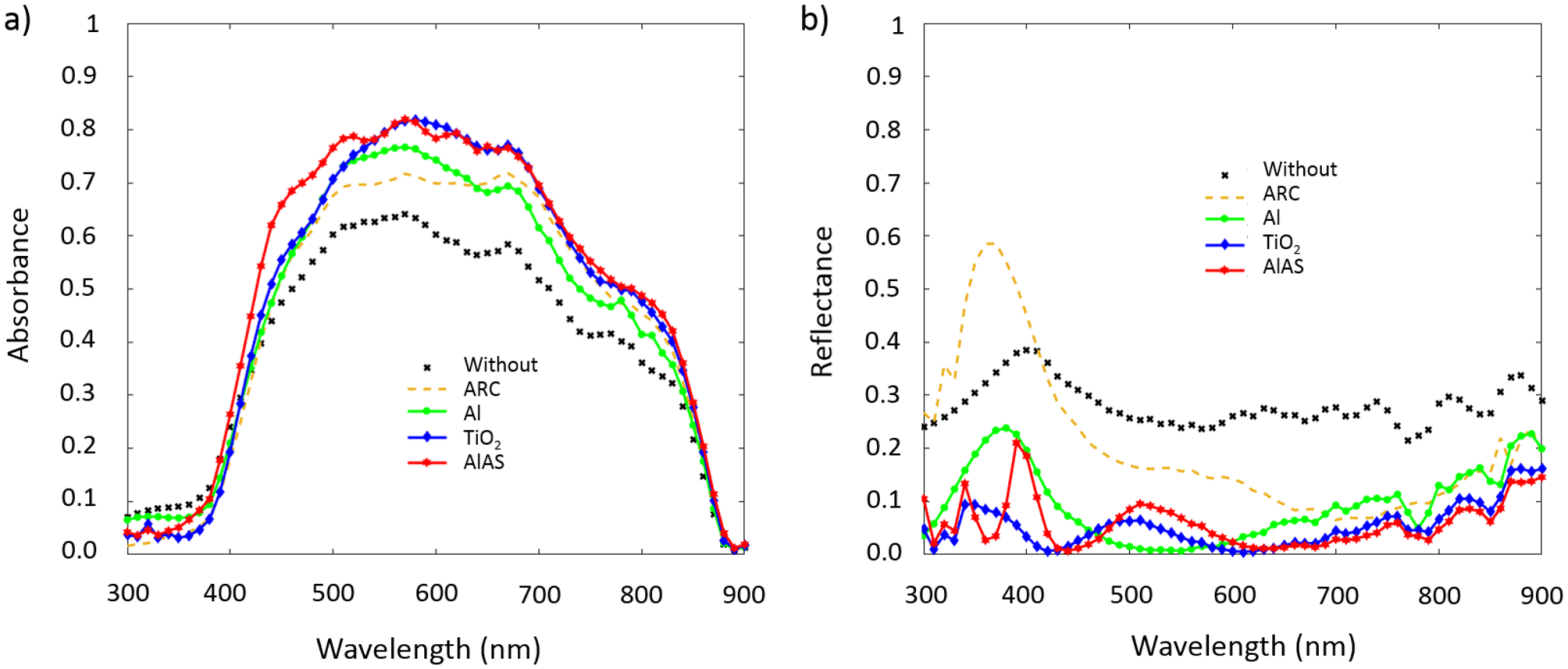
Spectral evolution of (**a**) the absorbance in the active layer and (**b**) the total reflectance of a GaAs solar cell for five different configurations: a conventional device without photonic strategies, a solar cell including an antireflection coating, a device with aluminum plasmonic nanoparticle on top of it and a solar cell including dielectric nanoparticle on it (TiO_2_ or AlAs).

Figure 2 shows both the absorbance in the active layer (Fig. 2a) and the total reflectance of the device (Fig. 2b), as a function of the incident wavelength, of a GaAs solar cell with different configurations and under a normal incidence. In particular, these figures show the cases of a conventional GaAs solar cell, a GaAs solar cell with an antireflection coating, a GaAs solar cell including plasmonic (Al) nanoparticles like in^31^, and a GaAs solar cell with dielectric nanoparticles on top of it made of either TiO_2_ or AlAs as proposed in this work. The resonant behavior of these dielectric nanoparticles produces a strong light confinement around them. Light is consequently reemitted towards their bottom part, thus reducing the reflectance and increasing the amount of light going inside the photovoltaic device. The material, size and arrangement of the nanoparticles allow us to select the bandwidth where these effects happen, accordingly matching it with the absorption window of the active material.

As it can be seen, all the considered cases provide an increase of the absorbance from 400 to 850 nm as well as a reduction of the total reflectance, maximizing the amount of light reaching inside the active layer, and consequently increasing the amount of photogenerated electron-holes pairs. However, the results of each technique are quite diverse. For instance, the inclusion of an ARC improves the optical absorbance mainly at large wavelengths (600–850 nm), as it can be seen in Fig. 2a. It also reduces the total reflectance in this spectral range, but its effects are remarkably worse at short wavelengths (Fig. 2b). On the other hand, the inclusion of resonant nanoparticles at the top part of the solar device provides an overall increase of the absorbance and a reduction of the total reflectance, compared to the conventional case. Nevertheless, we can still highlight remarkable differences between the metallic and dielectric cases.

Regarding the active absorbance, it can be seen that dielectric nanoparticles (TiO_2_ or AlAs) supporting Mie resonances^38,39^ provide a larger boost of the absorbance than that from plasmonic nanostructures (Al), and in a wider spectral range. It is worth mentioning that the case of AlAs nanoparticles provides the best results in terms of absorbance, mainly due to the improvement of the absorbance in the blue range (400–500 nm), with a value up to a 40% higher than the conventional one. In addition, the optical properties of these dielectric materials in the solar spectrum produce a noticeable lower light absorption than that of metals (due to a low imaginary part of their refractive index), strongly reducing the thermal effects produced in the case of Al nanoparticles. In the case of the total reflectance (Fig. 2b), the effects of both metallic and dielectric nanostructures are similar: a general reduction of the reflectance compared to the conventional solar cell. Even so, the effects of the dielectric particles are again slightly better than those of the metallic ones, in particular in the UV range (< 400 nm) and at large wavelengths (> 600 nm).

**Figure 3:**
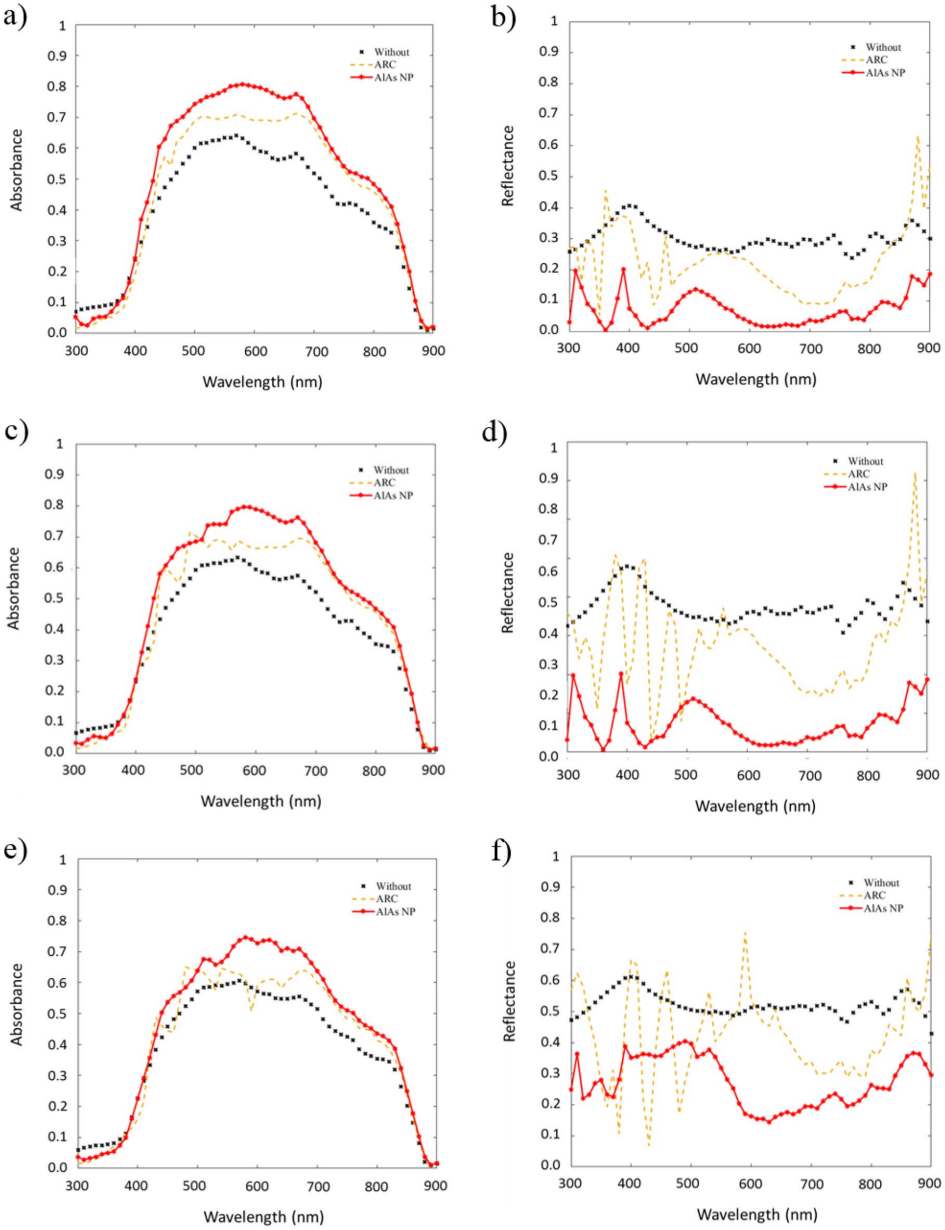
Absorbance in active layer (left) and total reflectance (right) of the device under an incidence of 20° in (**a**,**b**), of 40° in (**c**,**d**) and of 60° in (**e**,**f**).

While the maximum sensitivity of conventional solar cells is produced under a normal incidence, the addition of a textured or a nanostructured top surface may also increase the performance of the proposed device under non-normal angles of incidence. In this sense, Fig. 3 shows a comparison under different angles of incidence of the spectral evolution of both the absorbance (left panel) and the reflectance (right panel) of a conventional solar cell, a solar cell including an antireflection coating and our proposed device including dielectric nanoparticles on the top surface. Specifically, we consider an incidence of 20°, 40° and 60° as significant examples.

On the whole, the approach using AlAs nanoparticles provides the best results at any non-normal incidence in terms of both absorbance in the active layer and total reflectance. In the case of the absorbance (Fig. 3a,c,e), the use of these NPs in the top layer provides a smoother spectral response than the ARC case, as it will be explained, and similar to that of a conventional solar cell. Moreover, this absorbance is larger than that of the conventional one at any wavelength in the range between 400 nm and 850 nm. This enhancement is maintained for all the explored angles in the 500-800 nm range. The improvement only decays in the 400–550 nm range at high angles. The maximum increase appears at 440 nm and under normal incidence, being around a 44% compared to the conventional solar cell and a 33% to the case including an ARC. The maximum enhancement is shifted at larger wavelengths at high angular incidence. In this sense, under an incidence of 60° the maximum increase is estimated to be around 80% (630 nm) compared to the conventional cell and an 82% (600 nm) compared to the case including an ARC. This effect allows a better performance of the solar cell during a larger number of hours in real installations without using solar tracker systems, when the maximum intensity of the solar spectrum shifts at red during dawn. Indeed, it is directly related with the total reflectance response in those wavelength intervals (Fig. 3b,d,f), which spectral evolution shows that the minimum reflectance in the case of the solar cell with NPs is in the range between 600 and 700 nm. Again, the reflectance is much smaller within the solar spectrum than that of the other considered cases. In particular, this is clearly observed at incident angles of 20° and 40° (Fig. 3b,d). The case of 60° (Fig. 3f) is far complex because of the interferences produced in the multilayer ARC. Additionally, the differences between this minimum and the reflectance at other wavelengths are more prominent as the incident angle increases. It is also important to highlight that the reflectance peaks appear at ultraviolet wavelengths for the NPs case. These peaks become more noticeable and wider as the incident angle increases, producing a lower absorbance of this configuration in the spectral range (400–500 nm). However, these values are still better than those of a conventional solar cell.

In contrast to this response, the inclusion of ARC influences the normal incidence response at most. The extra layers produce remarkable interferential effects, in both the absorbance and the total reflectance, which are more conspicuous as the incident angle increases. This produces a response full of peaks—especially at low wavelengths, with mean values lower than the case of the solar cell with nanoparticles. Meanwhile, the behavior of the nanostructured solar cell is acceptable, keeping its effect on the improvement of absorbance within the solar spectrum.

**Figure 4:**
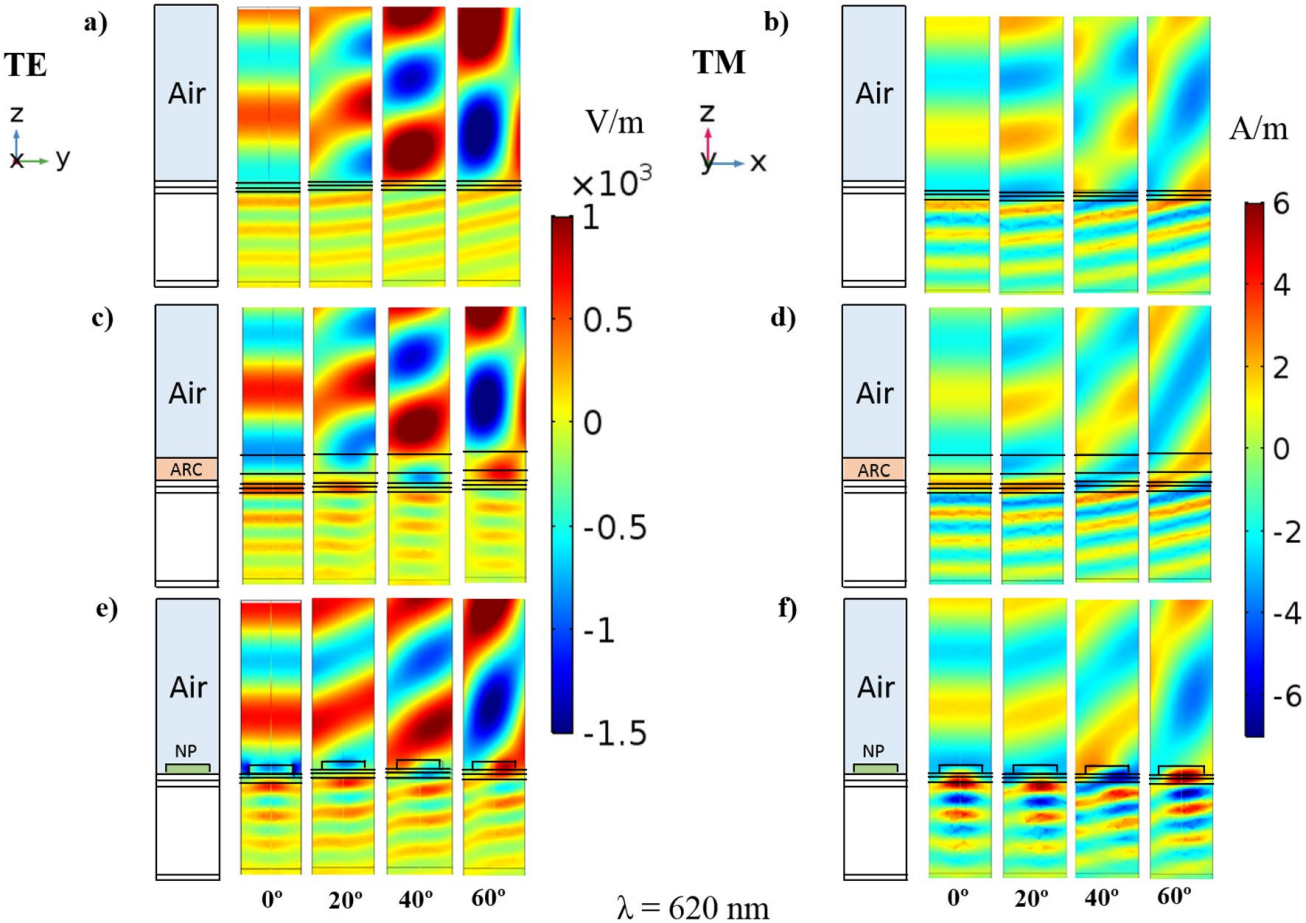
Spatial distribution of the electric and magnetic field intensities in the solar cell. (**a**) Electric field and (**b**) magnetic field distribution in a conventional solar cell. (**c**) Electric field and (**d**) magnetic field distribution in a GaAs solar cell including an ARC. (**e**) Electric field and (**f**) magnetic field distribution in a GaAs solar cell including AlAs nanoparticles on it. All the cases consider four different angular incidences, from left to right: normal (0°), 20°, 40° and 60°. The images show a unit cell of the device (w = 350 nm) with an air layer (1 μm) on top of it. Only the space (SiO_2_, 25 nm), window (InGaP, 30 nm) and GaAs (500 nm) layers are shown in the reference case. The considered ARC has a thickness of 120 nm and the AlAs nanoparticles have a diameter of 250 nm and a height of 50 nm.

In order to examine the origin of these effects, Fig. 4 shows the spatial distribution of the electric-field (left) and the magnetic-field (right) intensity inside the GaAs layer of the different solar devices that we are comparing. These images show the top region of a unit cell (w = 350 nm) of the device including an air layer of 1 μm, the space layer (SiO_2_, 25 nm), the window layer (InGaP, 30 nm) and the GaAs active layer (500 nm). The BSF layer and the substrate are not shown because the interesting behavior is produced in the GaAs layer. While Fig. 4a,b correspond to the conventional solar cell, Fig. 4c,d consider a GaAs solar cell with an ARC (120 nm) and Fig. 4e,f show the results of the GaAs solar cell with AlAs nanoparticles (diameter 250 nm and height 50 nm). All the figures are obtained at an incident wavelength of 620 nm (the one for the most remarkable differences in Fig. 3) and under four different incident angles, which are labeled on the bottom part of the figure. To clarify each structure a scheme of each cell configuration is included. It is clearly observed how the ARC (Fig. 4c,d) produces a larger concentration of light in the device than in the conventional case (Fig. 4a,b). This is more obvious in the layers that are above the active one, producing a certain reduction of the reflectance and an increase of the light reaching the active material. In contrast, the use of nanostructures on top of the device (Fig. 4e,f) provides a larger light confinement and also a more efficient guiding of light towards the active layer. In fact, it can be clearly observed that both the electric and the magnetic field are enhanced in this configuration compared to the previous cases. This phenomenon is mainly due to resonant effects of the nanoparticles with the incident field. The resonant nanoparticles efficiently confine the electromagnetic field and scatter it again towards the bottom part (the active region). Moreover, these effects are remarkably insensitive to the incident angle, providing better results than the other two considered cases. This shows up that this solution is a promising way to improve the optical performance of solar cells.

**Figure 5:**
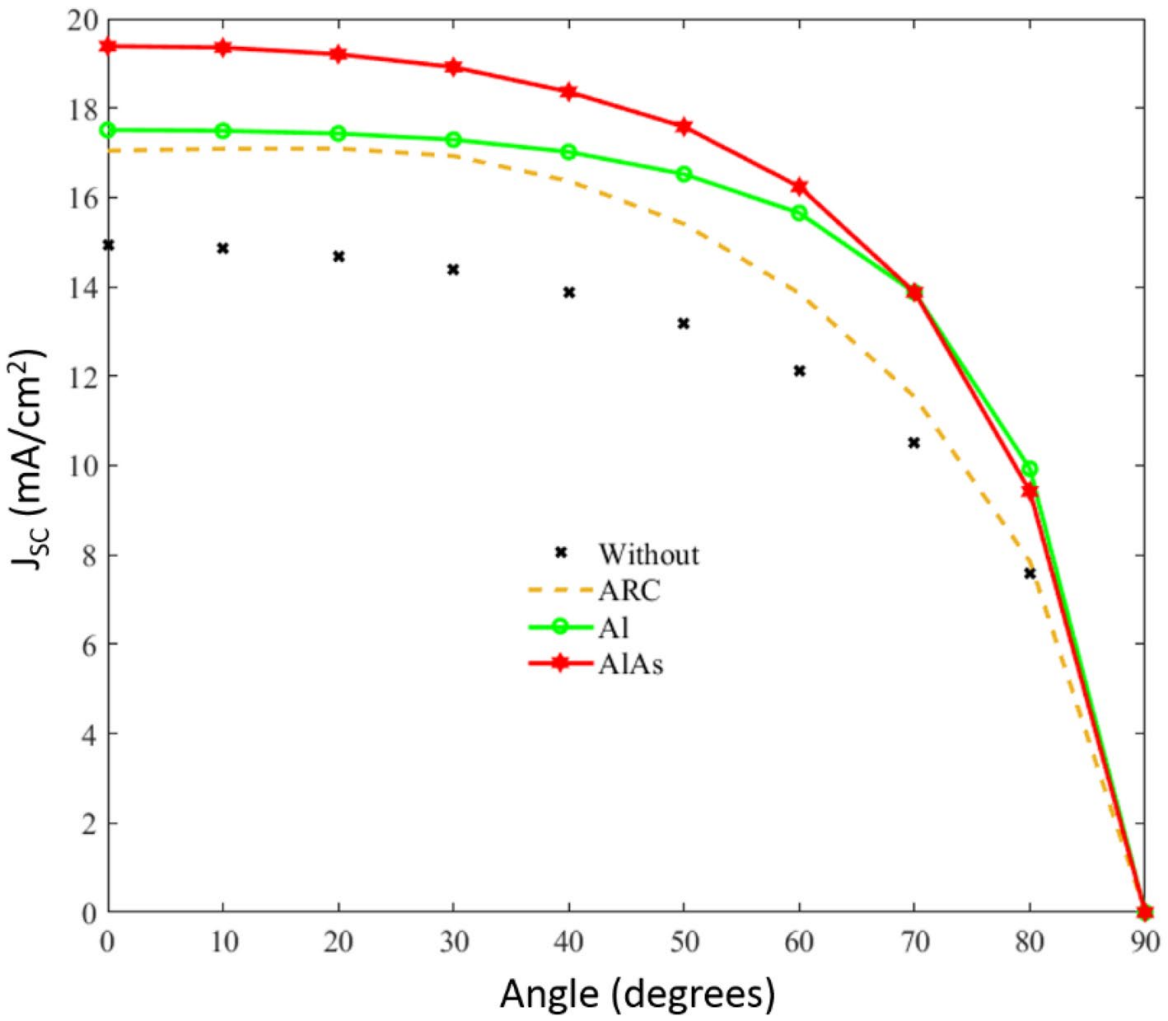
Short-circuit current density (J_SC_) as a function of the incident angle for the considered cases of a standard GaAs solar cell (x—none line), a GaAs with an ARC (dashed line) and a GaAs with nanoparticles on it. These nanoparticles are either metallic ones (o—solid line) like in ref^31^ or the proposed dielectric AlAs nanoparticles (hexagram—solid line).

Finally, Fig. 5 shows the simulated short-circuit current density (J_SC_) as a function of the incidence angle. As before and with the aim of comparing, this figure includes a conventional GaAs solar cell, the solar cell with an ARC and the solar cell with nanoparticles on top of it considering two cases: plasmonic Al nanoparticles, and the proposed dielectric AlAs nanoparticles. Again, it can be seen that although ARC improves the electric performance of a standard solar cell, the use of nanostructures on the top layer provides a better performance in a large angular range (J_SC_ is larger than 10 mA/cm^2^ up to 80° instead of the 70° angle of the ARC case). In this case, while metallic Al NPs previously proposed in the literature^31^, slightly improve the J_SC_ of an ARC solution, the proposed AlAs NPs significantly rise the current density in the angular range from 0° to 70°, producing a much better response of the solar cell than the other considered cases, also avoiding the thermal effects of the plasmonic nanoparticles.

## Conclusions

The aim of this work is focused on improving the optical response of GaAs solar cells using simple nanostructures (e.g. nanodisks and/or nanocylinders). Following the current state of the art and our previous works, we know that resonant nanoparticles, like plasmonic ones, can confine the incident electromagnetic field and scatter it into the bottom layer. Consequently, the amount of light inside the active layer increases, providing both an increase of the absorption and a decrease of the reflectivity of the solar device. However, the properties of these nanostructures, their fabrication and integration in the solar cell and its potential effect on the electric properties of the device should be carefully analyzed for every case in order to optimize the overall performance of the solar cell.

With this in mind, we have numerically analyzed the case of a GaAs-based solar cell, which use on space missions is remarkable. The proposed nanostructure is one of the most feasible arrangements from an experimental point of view, being composed of nanocylinders or nanodisks, depending on the aspect ratio. Additionally, the nanostructure is placed on the top of the device, simplifying its integration, making its fabrication more feasible, and reducing its influence on the electric properties of the solar cell. Furthermore, we have contrasted the numerical results with typical designs in the state-of-the-art, such as a conventional GaAs solar cell, a solar cell with an antireflection coating (ARC) and the solar device including plasmonic nanoparticles.

Our optical simulations shows that the use of AlAs nanoparticles is the best and simplest approach to efficiently confine the light, reducing the reflectance and increasing the absorbance of the solar device. This improvement compared to the other considered cases is observed all over the solar spectrum, and under a wide angular incidence. In fact, the spatial distribution of the electromagnetic field shows that these nanoparticles can guide the incident light into the active layer in a more effective way than an ARC. Specifically, it has been estimated that the short-circuit current density (J_SC_) using AlAs nanoparticles is 19.34 mA/cm^2^ at normal incidence. This is a relative increase of a 30% and a 21% when compared to a conventional GaAs solar cell and a GaAs solar cell with an ARC, respectively. Also, the dielectric nature of these nanoparticles avoids the thermal effects of the plasmonic nanoparticles (e.g. Al) which might involve harmful effects on the electric performance and/or the lifetime of the device.

In summary, this study reveals how the use of semiconductor nanoparticles can improve the optical performance of GaAs-based solar cells with no further antireflective coatings inclusion, improving the optical absorbance and reducing the reflectivity by means of light trapping effects, while avoiding unwanted thermal effects.

## Data Availability

The datasets generated and/or analysed during the current study are not publicly available because they have been generated using COMSOL Multiphysics, but are available from the corresponding author on reasonable request.
